# Polyhydramnios associated with rare genetic syndromes: two case reports

**DOI:** 10.1186/s13256-024-04435-0

**Published:** 2024-02-19

**Authors:** C. W. C. Lim, I. E. Lustestica, W. B. Poon, W. C. Tan

**Affiliations:** 1 Department of Obstetrics & Gynaecology, Singapore General Hospital, Singapore, Singapore; 2 Department of Neonatology & Developmental Medicine, Singapore General Hospital, Singapore, Singapore

**Keywords:** Genetics, Polyhydramnios, Kagami–Ogata syndrome, Grieg cephalopolysyndactyly syndrome, Amnioreduction, Polydactyly, Microcephaly

## Abstract

**Background:**

We present two genetic causes of polyhydramnios that were challenging to diagnose due to their rarity and complexity. In view of the severe implications, we wish to highlight these rare genetic conditions when obstetricians consider differential diagnoses of polyhydramnios in the third trimester.

**Case presentation:**

Patient 1 is a 34-year-old Asian woman who was diagnosed with polyhydramnios at 28 weeks’ gestation. First trimester testing, fetal anomaly scan, and intrauterine infection screen were normal. Subsequent antenatal ultrasound scans revealed macroglossia, raising the suspicion for Beckwith–Wiedemann syndrome. Chromosomal microarray analysis revealed a female profile with no pathological copy number variants. The patient underwent amnioreduction twice in the pregnancy. The patient presented in preterm labor at 34 weeks’ gestation but elected for an emergency caesarean section. Postnatally, the baby was noted to have a bell-shaped thorax, coat hanger ribs, hypotonia, abdominal distension, and facial dysmorphisms suggestive of Kagami–Ogata syndrome. Patient 2 is a 30-year-old Asian woman who was diagnosed with polyhydramnios at 30 weeks’ gestation. She had a high-risk first trimester screen but declined invasive testing; non-invasive prenatal testing was low risk. Ultrasound examination revealed a macrosomic fetus with grade 1 echogenic bowels but no other abnormalities. Intrauterine infection screen was negative, and there was no sonographic evidence of fetal anemia. She had spontaneous rupture of membranes at 37 + 3 weeks but subsequently delivered by caesarean section in view of pathological cardiotocography. The baby was noted to have inspiratory stridor, hypotonia, low-set ears, and bilateral toe polysyndactyly. Further genetic testing revealed a female profile with a pathogenic variant of the *GLI3* gene, confirming a diagnosis of Greig cephalopolysyndactyly syndrome.

**Conclusion:**

These cases illustrate the importance of considering rare genetic causes of polyhydramnios in the differential diagnosis, particularly when fetal anomalies are not apparent at the 20-week structural scan. We would like to raise awareness for these rare conditions, as a high index of suspicion enables appropriate counseling, prenatal testing, and timely referral to pediatricians and geneticists. Early identification and diagnosis allow planning of perinatal care and birth in a tertiary center managed by a multidisciplinary team.

## Background

Polyhydramnios is a medical condition characterized by an excessive accumulation of amniotic fluid within the amniotic sac during pregnancy. It typically occurs in approximately 1–2% of pregnancies and can result from multiple causes [1]. It can be idiopathic, due to maternal conditions such as diabetes mellitus, fetal anomalies, multiple pregnancies, or genetic abnormalities [2]. The excessive amniotic fluid can lead to an increased risk of complications such as preterm labor, fetal malpresentation, placental abruption, and postpartum hemorrhage [3]. Polyhydramnios can be categorized as mild, moderate, or severe on the basis of the amniotic fluid index or deepest vertical pocket measurements [4]. The more severe the polyhydramnios, the higher the risk of fetal morbidity [5].

Maternal diabetes and structural abnormalities that affect fetal swallowing as well as amniotic fluid stasis typically account for the more common causes of polyhydramnios [6]. Chromosomal abnormalities and genetic conditions must be considered in fetuses with polyhydramnios; however, the prenatal diagnosis of genetic conditions is extremely challenging [7], especially if the genetic condition in question is rare. In this article, we present two rare genetic causes of polyhydramnios that were diagnosed postnatally. As the genetic conditions described forthwith in this article have variable implications on physical, social, and functional development of the infant, we wish to highlight polyhydramnios as a presenting feature of these genetic conditions to raise awareness among obstetricians with an aim toward early intervention and informed decision-making.

## Case presentation

### Case 1

**Fig. 1:**
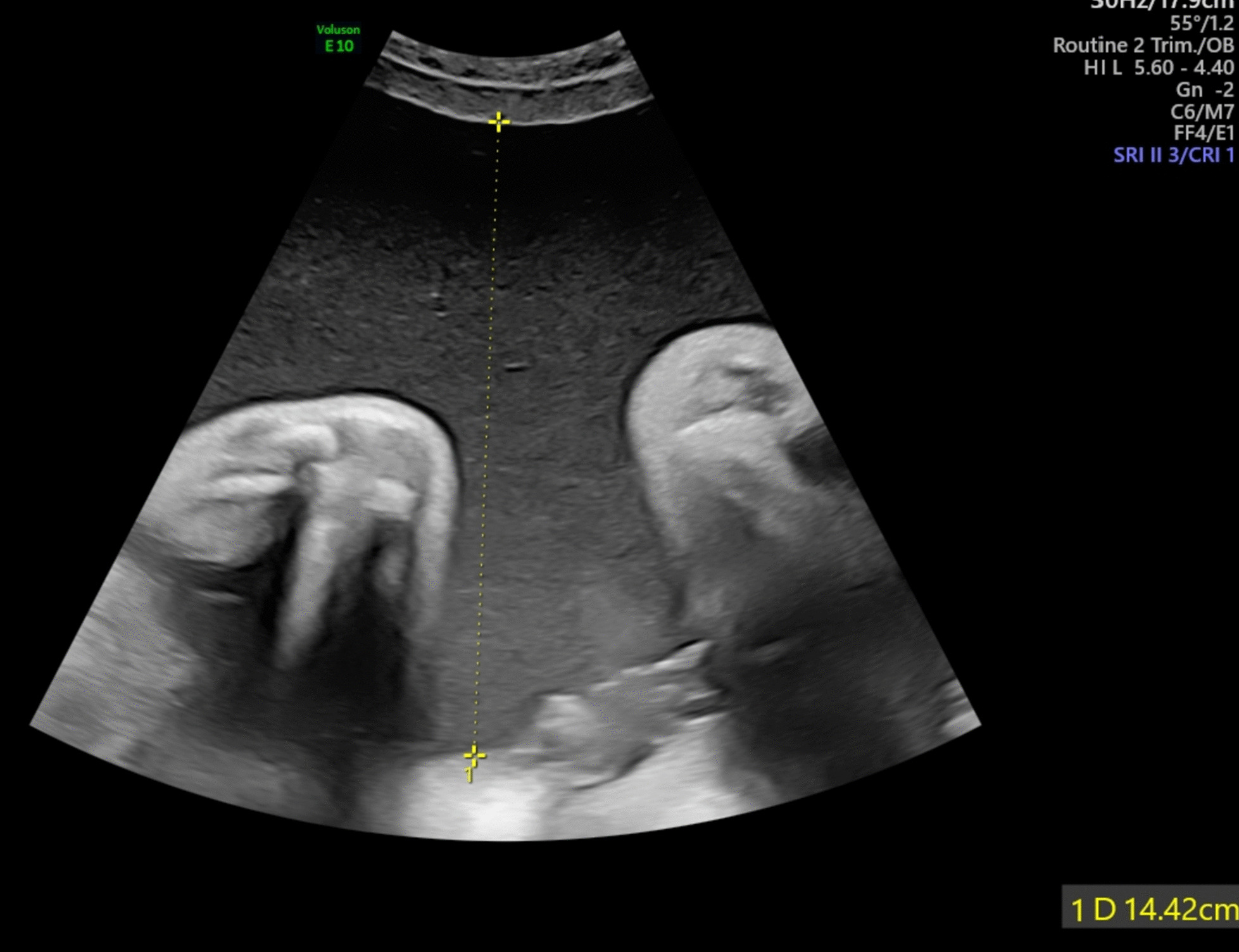
Ultrasound at 31 weeks showing deepest vertical pocket of 14 cm

**Fig. 2:**
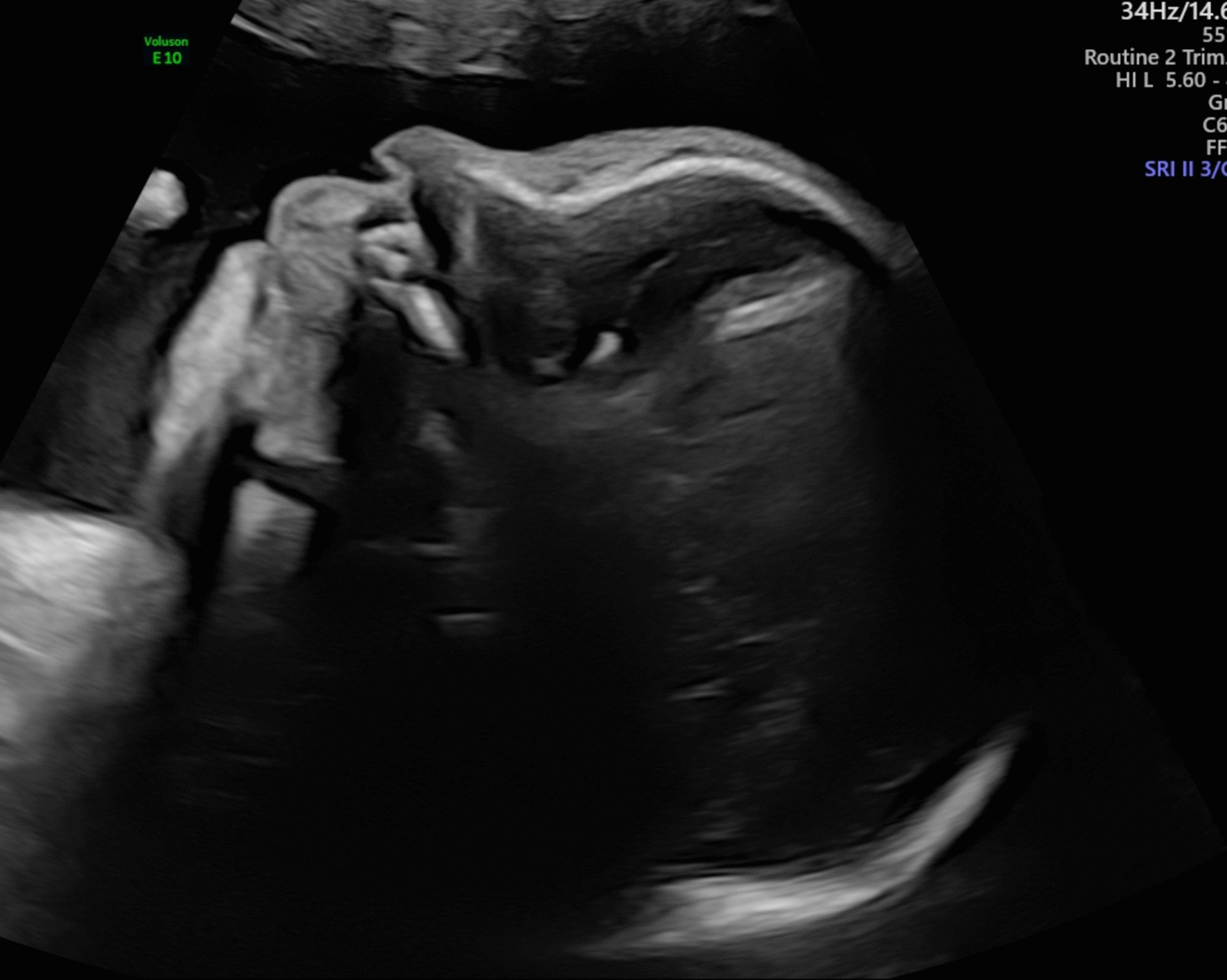
Ultrasound at 31 weeks showing micrognathia

Patient 1 is a 34-year-old Asian woman, gravida 2 para 1, who was diagnosed with polyhydramnios at 28 weeks of pregnancy. She had a term uncomplicated delivery in 2017 with no background history of gestational diabetes, pre-eclampsia, or any adverse perinatal events. She booked early at 10 weeks for antenatal care. Routine antenatal serologies and blood tests were normal. First-trimester screening was low risk for trisomies 21, 18, and 13, and nuchal translucency was within normal limits. Screening scan at 20 weeks of gestation did not show any structural abnormalities. Amniotic fluid index (AFI) was normal with estimated fetal weight at the 80th centile. A 3-point oral glucose tolerance test was normal. Patient 1 complained of abdominal discomfort at 28 weeks of gestation, when severe polyhydramnios (AFI 40 cm) was diagnosed. Intrauterine infection screen for toxoplasmosis, cytomegalovirus, and parvovirus B19 were negative. In view of persistent symptomatic polyhydramnios, she underwent amnioreduction at 29 and 31 weeks of gestation when amniotic fluid deepest vertical pocket (DVP) reached 14 cm (Fig. 1) with cervical funneling. Karyotype and chromosomal microarray analysis (CMA) revealed a female profile with no pathological copy number variants (CNVs). Subsequent ultrasound scans of the fetus revealed macroglossia and micrognathia (Fig. 2). No abdominal wall defects were detected. AFI and Dopplers were monitored weekly. Intramuscular bethamethasone was administered, and the patient was counseled by the neonatology team. The patient presented in pre-term labor at 34 weeks of gestation but opted for an emergency caesarean section.

**Fig. 3:**
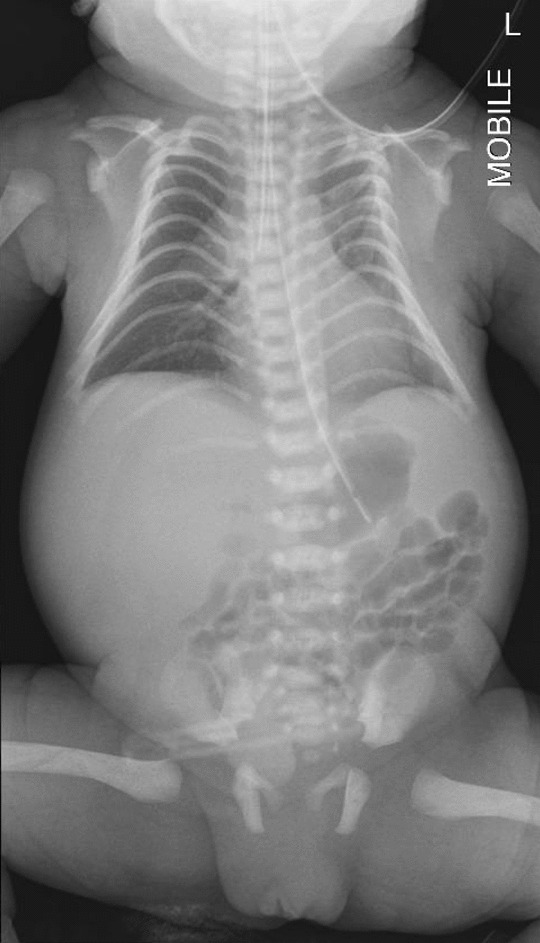
Chest X-ray showing “coat hanger” ribs

A baby girl weighing 2580 g was delivered at 34 weeks of gestation with APGAR scores 6 and 7. Intermittent positive pressure ventilation (IPPV) was administered in view of poor respiratory effort at birth. The baby was noted to have a distended abdomen, poor air entry, and small lung volume. Intubation of the infant was difficult due to macroglossia and an anteriorly displaced larynx. Further testing was performed on the basis of a clinical suspicion of Kagami–Ogata syndrome (KOS), due to the presence of suggestive features such as facial dysmorphisms (elongated philtrum; small, low-set ears; hypertelorism; short neck; micrognathia; microstomia; macroglossia; and a tiny anterior fontanelle), coat-hanger ribs (Fig. 3), diastasis recti, hypotonia, and poor respiratory and swallowing effort. Genetic testing confirmed hypermethylation of the *MEG3* promoter—a specific imprinting defect associated with KOS. Baby 1 was hospitalized for almost 3 months and discharged home with continuous positive airway pressure (CPAP) breathing support and nasogastric tube feeding. She is currently on follow-up with the multidisciplinary complex care team and requires follow-up visits at least every 3 months for the next 8 years.

### Case 2

Patient 2 is a 30-year-old Asian primiparous patient with a history of subfertility who booked at 9 weeks’ gestation following conception via frozen embryo transfer. She had no background medical history. Routine antenatal serologies and blood tests were normal. First-trimester ultrasound revealed a nuchal translucency above the 95th centile. The patient declined invasive genetic testing, and non-invasive prenatal testing (NIPT) was low risk. Her fetal anomaly scan at 18 weeks did not show any structural abnormalities. She developed herpes zoster at 19 weeks of gestation, which resolved after 1 week of oral acyclovir. The fetus was noted to have grade 1 echogenic bowels at 23 weeks gestation. Intrauterine infective screen for toxoplasmosis, cytomegalovirus, and parvovirus B19 were negative. She was diagnosed with polyhydramnios at 27 weeks of gestation with a deepest vertical pocket of 8 cm, which increased thereafter to 10 cm by 35 weeks. Patient 2 did not require amnioreduction and was asymptomatic except for abdominal bloating. Serial Doppler scans did not reveal growth restriction or sonographic evidence of anemia. She presented with spontaneous rupture of membranes at 37 weeks and 4 days of gestation and subsequently underwent an emergency caesarean section for fetal distress.

**Fig. 4:**
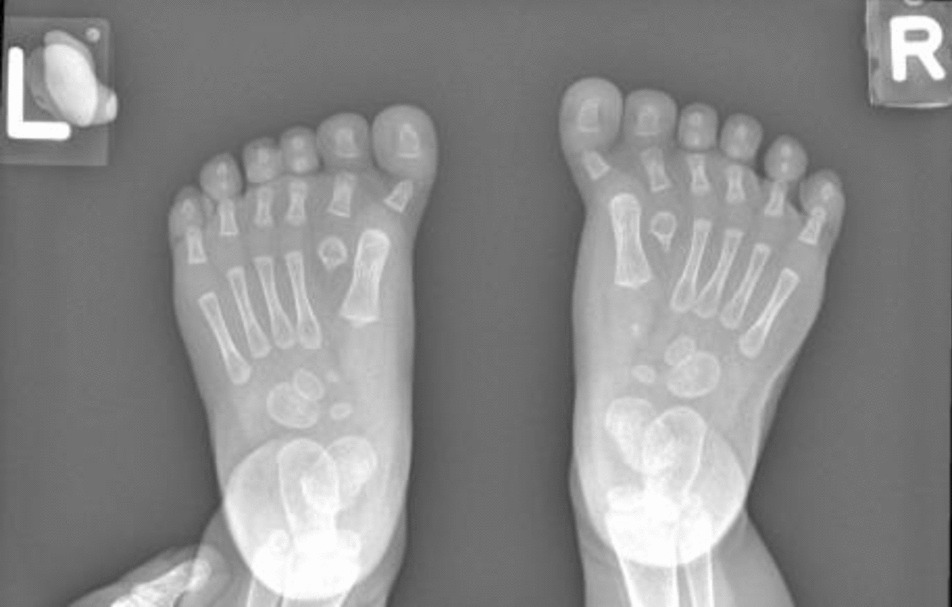
X-ray showing bilateral big toe polydactyly

**Fig. 5:**
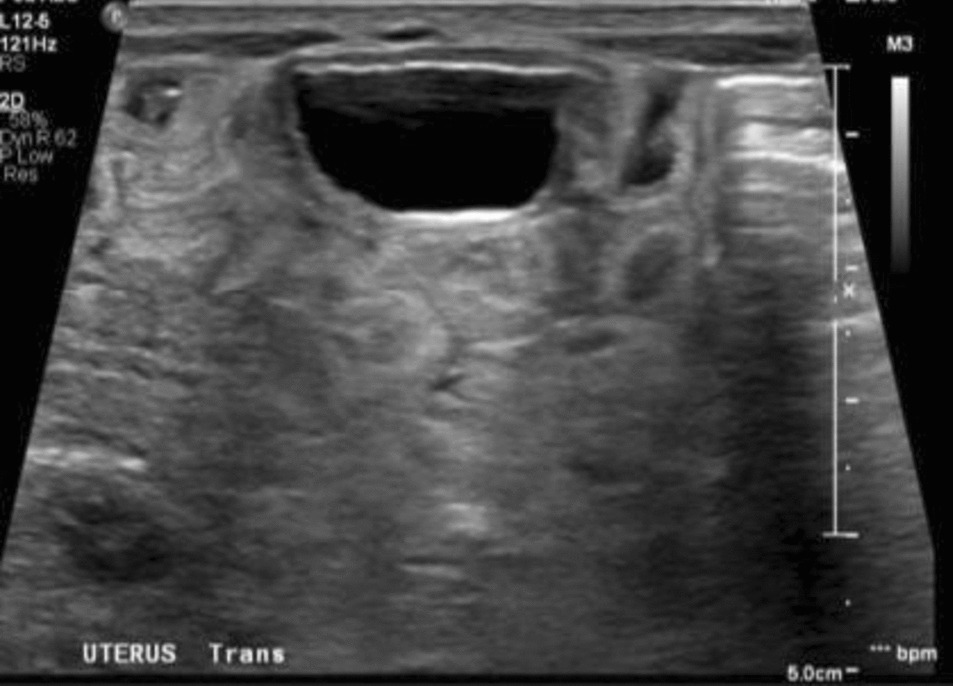
Abdominal ultrasound showing bicornuate uterus

**Fig. 6:**
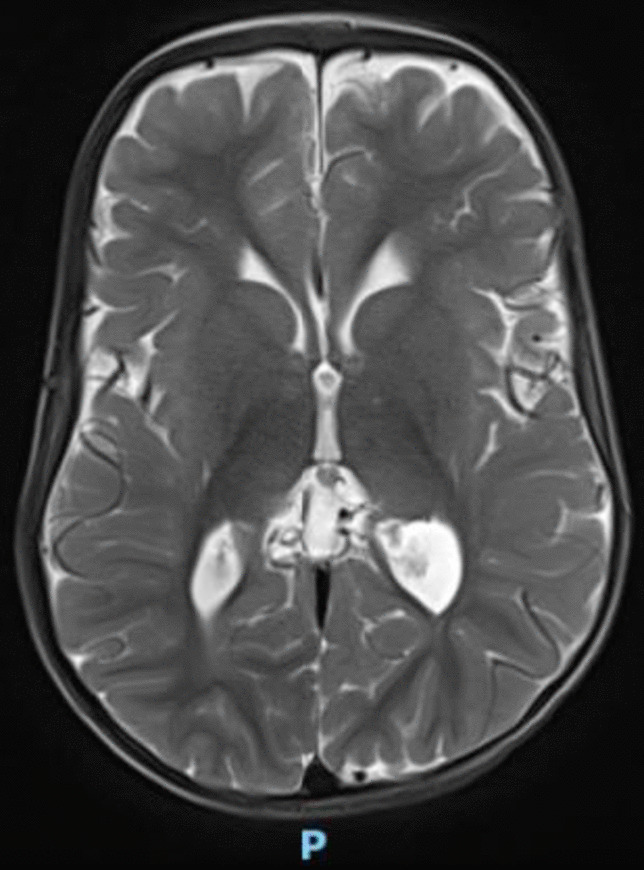
Normal magnetic resonance imaging of the brain

Baby 2, weighing 3150 g, was born with APGAR scores 5 and 9. At birth, she presented with hypotonia and inspiratory stridor on handling. The baby required IPPV at birth but was subsequently weaned off to room air. Clinical features included dolichocephaly, flat nasal bridge, low-set ears, bilateral simian creases, bilateral genu recurvatum, bilateral big toe polydactyly (Fig. 4), and syndactyly of the second and third toes. Hyperextension of both knees with ventral position of the tibiae, bicornuate uterus (Fig. 5), and mild tracheomalacia were subsequently confirmed. No intracranial anomalies were detected on intracranial imaging (Fig. 6). CMA showed a female profile with a pathogenic variant of the *GLI3* gene, confirming the diagnosis of Greig cephalopolysyndactyly syndrome.

Baby 2 was managed by a multidisciplinary team of specialists (neonatology, genetics, pediatric orthopedics, otorhinolaryngology) and allied health professionals (physiotherapy and speech therapy). Upon discharge, the infant remained under long-term care with her primary neonatologist and enrolled in an early intervention programme for global developmental delay. She had frequent hospital admissions for recurrent bronchiolitis in her first year of life. At 1 year and 8 months of age, Baby 2 had severe delays in receptive and expressive language, preverbal skills, and motor developmental delays and remains on follow-up with physiotherapy and speech and language therapy.

## Discussion

In this case series, we presented two rare genetic causes of polyhydramnios that were diagnosed postnatally. In the existing literature, the risk of postnatal diagnosis of genetic abnormalities in a pregnancy with idiopathic polyhydramnios can be as high as 28% if the infant received long-term follow-up thereafter [8]. Current evidence [8] does not support routine amniocentesis for isolated polyhydramnios in the absence of other key structural defects. However, as genetic abnormalities have long-lasting impact on the newborn infant, early diagnosis is pertinent to reducing the need for extensive evaluations and to improve the outcomes of the neonate [9]. We would like to henceforth highlight the features of these two rare genetic conditions.

Kagami–Ogata syndrome (KOS) is a rare entity with severe prognosis caused by a genetic imprinting disorder leading to polyhydramnios, facial dysmorphisms, and skeletal abnormalities—most classically “coat hanger ribs”, respiratory distress, abdominal wall defects, fetal macrosomia, developmental delay and intellectual disability [10]. A case series of Japanese patients [11] diagnosed with Kagami–Ogata syndrome lists polyhydramnios, placentomegaly, and abdominal wall defects (for example, omphalocele) as characteristic features of KOS. As described in our first patient, Patient 1, she was noted to have severe polyhydramnios, fetal macroglossia, and micrognathia and was counseled about Beckwith–Wiedemann syndrome as a differential diagnosis. A case report from Spain [12] described the prenatal diagnosis of KOS in view of several unifying features picked up on antenatal scans—severe polyhydramnios and a bell-shaped thorax as well abdominal wall defect. A 3D ultrasound also demonstrated characteristic facial dysmorphisms such as retrognathia, bulky cheeks, and protruding philtrum. In contrast, our patient manifested only polyhydramnios and micrognathia as features of KOS. While characteristic, these are not specific to KOS. The use of fetal magnetic resonance imaging (MRI) and 3D ultrasound in the calculation of coat-hanger angle and thoracic ratios as a diagnostic adjunct to the typical sonographic features of abdominal wall defects and polyhydramnios has also been described in the literature [13]. Once common causes of polyhydramnios have been excluded, the presence of abdominal wall defects and bell shaped thorax should lead the clinician to suspect KOS. As the prognosis of KOS is poor [11], prenatal diagnosis is essential and enables parents to make informed decisions regarding the course of the pregnancy and to prepare for the eventual care of the infant.

Greig cephalopolysyndactyly syndrome (GCPS) is a rare genetic disorder characterized by physical abnormalities affecting the digits and the craniofacial area. Its incidence is estimated to be 1–9 in a million [14]. Craniofacial malformations include a large skull, frontal bossing, broad nasal bridges, and hypertelorism, while digital abnormalities include extra fingers or toes or cutaneous or osseous syndactyly [15]. It is an autosomal dominant inherited condition caused by a mutation of the gene *GLI3* located on chromosome 7 [16]. As far as we know, our patient is the first who presented with polyhydramnios, albeit a mild form, that did not cause cervical shortening nor needed amnioreduction. A case report from Spain described the prenatal diagnosis of GCPS [17] in view of sonographic features of polydactyly, bilateral ventriculomegaly, and macrocephaly as well as hypertelorism. In contrast to the case we have reported, our patient’s fetal anomaly scan only manifested echogenic bowels in the absence of the classical features of GCPS, with polyhydramnios diagnosed in the third trimester. The clinical prognosis of GCPS and life expectancy is comparable to the general population [17]. Skeletal abnormalities, as in the case with GCPS, may be amenable to surgical correction with good functional outcomes [16].

For both our patients, polyhydramnios was the only apparent sonographic abnormality. A detailed anatomical survey of the fetus is thus essential to elicit other sonographic signs of associated genetic conditions. The Society of Maternal Fetal Medicine has published a suggested checklist [8] of the parameters and anatomical structures to focus on when severe polyhydramnios is diagnosed in addition to routine fetal growth, in which special attention is recommended to be accorded in a systematic fashion to the fetal facial structures, neck, heart, stomach, kidneys, and lower spine as well as positioning of the fetal limbs and fetal movement.

## Conclusion

These cases illustrate the importance of considering rare genetic causes of polyhydramnios in the differential diagnosis, particularly when there are accompanying fetal anomalies although most are subtle. We hope to highlight these rare conditions, as a high index of suspicion enables appropriate counseling, prenatal testing, and timely referral to pediatricians and geneticists. Early identification and diagnosis allow planning of perinatal care and birth in a tertiary center managed by a multidisciplinary team.

## Data Availability

All information about the patients in this case report come from the Department of Obstetrics & Gynaecology and Department of Neonatology at Singapore General Hospital. Data sharing is not applicable to this article, as no datasets were generated.

## Abbreviations

CMA: Chromosomal microarray analysis
CNV: Copy number variants
NIPT: Non-invasive prenatal testing
AFI: Amniotic fluid index
NICU: Neonatal intensive care unit
KOS: Kagami–Ogata syndrome
CPAP: Continuous positive airway pressure
DVP: Deepest vertical pocket
GCPS: Greig cephalopolysyndactyly syndrome
MRI: Magnetic resonance imaging
